# Compound C prevents Hypoxia-Inducible Factor-1α protein stabilization by regulating the cellular oxygen availability via interaction with Mitochondrial Complex I

**DOI:** 10.1186/1756-0500-4-117

**Published:** 2011-04-12

**Authors:** Yee Liu Chua, Thilo Hagen

**Affiliations:** 1 Department of Biochemistry, Yong Loo Lin School of Medicine, National University of Singapore, 117597 Singapore

## Abstract

The transcription factor Hypoxia-Inducible Factor-1α is a master regulator of the cellular response to low oxygen concentration. Compound C, an inhibitor of AMP-activated kinase, has been reported to inhibit hypoxia dependent Hypoxia-Inducible Factor-1α activation via a mechanism that is independent of AMP-activated kinase but dependent on its interaction with the mitochondrial electron transport chain. The objective of this study is to characterize the interaction of Compound C with the mitochondrial electron transport chain and to determine the mechanism through which the drug influences the stability of the Hypoxia-Inducible Factor-1α protein.

We found that Compound C functions as an inhibitor of complex I of the mitochondrial electron transport chain as demonstrated by its effect on mitochondrial respiration. It also prevents hypoxia-induced Hypoxia-Inducible Factor-1α stabilization in a dose dependent manner. In addition, Compound C does not have significant effects on reactive oxygen species production from complex I via both forward and reverse electron flux.

This study provides evidence that similar to other mitochondrial electron transport chain inhibitors, Compound C regulates Hypoxia-Inducible Factor-1α stability by controlling the cellular oxygen concentration.

## Findings

Compound C has been reported to inhibit hypoxia dependent Hypoxia-Inducible Factor-1α (HIF-1α) stabilization by interacting with the mitochondrial electron transport chain (ETC) and suppressing mitochondria generated reactive oxygen species [1]. This finding coincides with the hypothesis that increased reactive oxygen species (ROS) released from mitochondrial complex III during hypoxia stabilize HIF-1α [2-4]. However our recent findings showed that the mitochondrial electron transport chain controls the stability of HIF-1α during hypoxia independently of reactive oxygen species production [5]. We therefore studied the mechanism through which Compound C interacts with the ETC in detail.

### Effect of Compound C on hypoxia-induced HIF-1α protein stabilization

**Figure 1:**
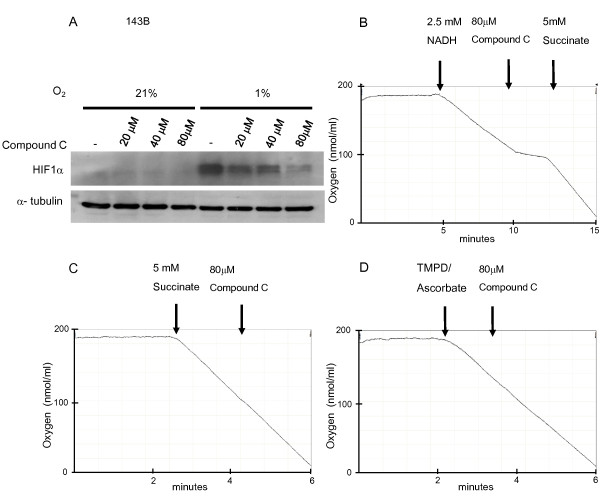
**Compound C acts as an inhibitor of mitochondrial complex I and inhibits hypoxia-dependent HIF-1 α stabilization**. A. 143B cells were incubated at 1% O_2 _for 4 hours in the presence of Compound C (20 μM, 40 μM and 80 μM). Whole cell lysates were separated by 10% SDS gel and probed for HIF-1α. B-D. Mouse liver mitochondria were isolated by differential centrifugation in mitochondrial isolation buffer containing 280 mM sucrose, 10 mM Tris (pH7.4) and 1 mM EDTA as described in [8] and subjected to one freeze thaw cycle before measurement. Oxygen consumption of mouse liver mitochondria was measured with 2.5 mM NADH, 5 mM succinate or 0.2 mM TMPD/1 mM ascorbate as respiratory substrates and 80 μM Compound C in mitochondrial isolation buffer using a Clark-type oxygen electrode at 37°C. The results shown are representative of 2 independent experiments.

We first determined the effect of Compound C on hypoxia-induced HIF-1α protein accumulation. 143B cells from ATCC (ATCC number: CRL-8303) were incubated at either 21% or 1% O_2 _for 4 hours in the presence of Compound C (20 μM, 40 μM and 80 μM) and then analyzed using immunoblotting. At 1% O_2_, HIF-1α protein is stabilized in untreated control cells. This is due to inhibition of prolyl hydroxylases which in the presence of oxygen hydroxylate HIF-1 α and therefore target it for proteasomal degradation. The treatment with increasing concentrations of Compound C at 1% O_2 _caused a dose dependent decrease in HIF-1 α protein accumulation (Figure 1A). The result confirms that Compound C inhibits hypoxia induced HIF-1 α protein stabilization [1].

### Effect of Compound C on mitochondrial respiration

To study the effects of Compound C on the mitochondrial ETC, we measured the oxygen consumption in 0.5 mg of mouse liver mitochondria with a Clarke-type oxygen electrode. Mitochondria were subjected to one freeze-thaw cycle before the measurement and NADH (2.5 mM) was used as the respiratory substrate for complex I. Treatment of mitochondria with 80 μM Compound C inhibited respiration by 74.5%. Oxygen consumption resumed when the respiratory substrate for complex II, succinate (5 mM) was added, as illustrated in Figure 1B. These results suggest that Compound C is an inhibitor of complex I and does not inhibit downstream complexes. To determine if Compound C interacts with complex II, III or IV, Compound C was added to mitochondria respiring on succinate. Compound C did not inhibit succinate dependent oxygen consumption (Figure 1C) and therefore does not interact with complex II, III or IV. To directly test if Compound C is an inhibitor of complex IV, 0.2 mM 2,2,4-trimethyl-1,3-pentanediol (TMPD)/1 mM ascorbate was added. TMPD is an artificial electron donor that transfers electrons from ascorbate to complex IV via cytochrome *c*. Compound C had no effect on the oxygen consumption when TMPD/ascorbate was used as the respiratory substrate (Figure 1D). These results imply that Compound C inhibits mitochondrial respiration through its interaction with complex I.

### Effect of Compound C on ROS production from isolated mouse liver mitochondria

**Figure 2:**
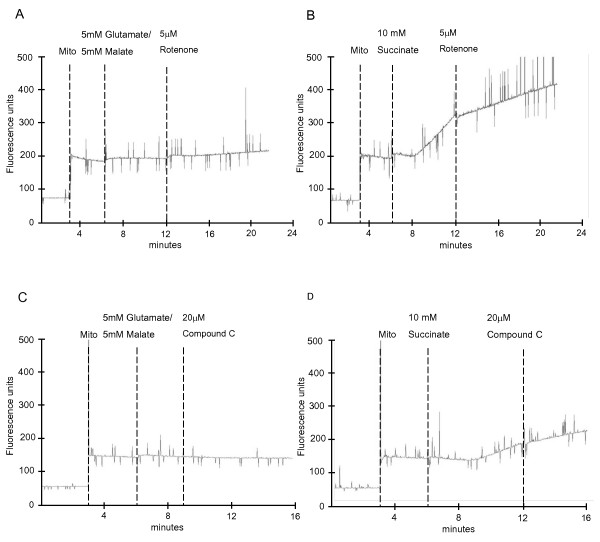
**Compound C does not increase hydrogen peroxide production from both forward and reverse electron flux of complex I**. 0.1 mg/ml of isolated mouse liver mitochondria were incubated in reaction buffer with either 5 mM glutamate/5 mM malate or 10 mM succinate as respiratory substrates or inhibitors (5 μM rotenone and 20 μM Compound C) at 37°C. Hydrogen peroxide production was assessed by homovanillic acid/horseradish peroxidase. Mitochondria, substrates and inhibitors were sequentially added as shown in the figure. The results shown are representative of 2 independent experiments.

Mitochondrial respiratory complex I is one of the two main sites that are capable of producing ROS within the ETC [6]. ROS from complex I can be derived from both forward and reverse electron flux. To determine whether ROS are produced from either of the electron fluxes in the presence of Compound C, the rate of hydrogen peroxide (H_2_O_2_) production was measured. In this assay, 0.1 mg/ml of isolated mouse liver mitochondria were added to 2 ml of reaction buffer (containing 125 mM KCl, 2 mM KH_2_PO_4_, 1 mM MgCl_2, _20 mM HEPES, 0.1 mM EGTA pH7.4) in the presence of 0.1 mM homovanillic acid, 0.5 μM horseradish peroxidase and different respiratory substrates at 37°C. The rate of H_2_O_2 _production was measured fluorometrically at Ex/Em 312/420 nm using a Perkin Elmer LS-55 fluorescence spectrometer. In the presence of glutamate and malate, addition of the well-established complex I inhibitor rotenone resulted in a moderate increase in the H_2_O_2 _production from complex I via the forward electron flux (Figure 2A). As expected, H_2_O_2 _production via reverse electron flux was inhibited by rotenone when the mitochondria were energized with succinate (Figure 2B). Addition of Compound C was without effect on H_2_O_2 _production via the forward electron flux (Figure 2C). H_2_O_2 _production via the reverse electron flux was also not increased but showed a slight decrease (Figure 2D).

To measure the cellular H_2_O_2 _and superoxide production in mammalian cells, fluorescence of the 2',7'-dichlorofluorescein (DCFDA) and dihydroethidium (DHE) dye, respectively, were monitored by flow cytometry. No significant difference in both the H_2_O_2 _and superoxide concentration was observed in cells treated with Compound C (at concentrations up to 100 μM) compared with untreated cells (data not shown). Taken together, the results suggest that Compound C mediated inhibition of hypoxia dependent HIF-1α stabilization is not due to inhibition of complex III derived ROS as previously suggested [1] but likely due to an effect on mitochondrial oxygen consumption.

## Conclusions

The aim of this study was to determine the mechanism through which Compound C inhibits the stabilization of the HIF-1 α protein in hypoxia. We provide evidence that Compound C acts as a complex I inhibitor that affects the stabilization of the HIF-1 α protein independently of ROS production. Our results suggest that Compound C, like other mitochondrial ETC inhibitors, regulates HIF-1 α stability by controlling the cellular oxygen concentration leading to increased cellular oxygen availability and reactivation of prolyl hydroxylases in hypoxia [5,7].

## Competing interests

The authors declare that they have no competing interests.

## Authors' contributions

YL carried out the experimental work and drafted the manuscript while TH participated in the design of the study, in evaluation of the results, in drafting and finalizing the manuscript. The authors have read and approved the final manuscript.
